# Bidirectional Causal Effect Between Gut Microbiota and Glioma Risk: A Systematic Review‐Based Mendelian Randomization and Immune‐Mediated Effect Analysis

**DOI:** 10.1002/cai2.70039

**Published:** 2025-12-09

**Authors:** Jiachen Wang, Yilin Zhang, Zhuang Kang, Shenglan Li, Rong Zhang, Mengqian Huang, Chengzhuo Wang, Yuxiang Fan, Xinrui Liu, Yuxiao Chen, Tingrui Han, Yuji Wang, Wenbin Li

**Affiliations:** ^1^ Department of Neuro‐oncology, Cancer Center, Beijing Tiantan Hospital Capital Medical University Beijing China; ^2^ Capital Medical University Beijing China; ^3^ Department of Medicinal Chemistry, College of Pharmaceutical Sciences Capital Medical University Beijing China; ^4^ Department of Neurosurgery, Beijing Tiantan Hospital Capital Medical University Beijing China; ^5^ Department of Neurosurgery, Xuanwu Hospital Capital Medical University Beijing China; ^6^ The First Clinical College of Capital Medical University, Xuanwu Hospital Capital Medical University Beijing China

**Keywords:** genome‐wide association study, glioma, gut microbiota, immune cell, Mendelian randomization

## Abstract

**Background:**

Glioma is the most common malignant tumor in the central nervous system, with unclear pathogenesis and poor treatment outcomes. Recent research reveals that the brain–gut axis—involving gut microbiota and immune activity—influences central nervous system tumors. Given the pivotal role of the brain–gut axis in glioma, our study aimed to elucidate the causal association between gut microbiota and glioma, and to identify potential immune‐mediated effects and therapeutic targets.

**Methods:**

Based on publicly available genome‐wide association study data, our research employed multi‐subgroup, replicated, Bayesian weighted, and summary statistics‐based two‐sample Mendelian randomization (MR) studies, combined with the Preferred Reporting Items for Systematic Reviews and Meta‐Analyses (PRISMA) systematic review strategy, to systematically evaluate the potential causal effects of gut microbiota on glioma and their immune‐mediated traits.

**Results:**

The initial screening identified 53 gut microbiota and 58 plasma immune traits with potential causal associations with glioma. Through external data and systematic review from six studies, we ultimately confirmed five gut microbiota‐plasma immune trait‐glioma pathways. CD28^+^CD45RA^−^ CD8dim Treg (OR = 0.019, *p* = 0.007) mediated the risk of Bacteroides A plebeius A (OR = 0.149, *p* = 0.036) on glioma, accounting for 2.99% of the effect; the proportion of CD4^+^ memory T cells in whole blood (OR = 0.066, *p* = 0.029) mediated the risk of Bacteroides sp002160055 (OR = 0.158, *p* = 0.024) on non‐glioblastoma(GBM), accounting for 8.51% of the effect, while the risk of Faecalicoccus (OR = 0.345, *p* = 0.005) on non‐GBM was jointly mediated by the absolute number of Naive CD8br and the expression of CD19 in IgD^+^ CD38br B cells. The protective effect of Faecalibacterium sp002160895 on GBM was mediated by 7.59% of the expression level of CD4 in Treg cells.

**Conclusion:**

Our study, through MR analysis, revealed the causal relationship between gut microbiota and the susceptibility to glioma, and for the first time proposed the important role of circulating immune cells in this process, providing new potential biomarkers for the early diagnosis and treatment of glioma.

## Graphical abstract

Through Mendelian randomization analysis, the study confirmed a causal relationship between the gut microbiota and the risk of glioma. The most significant finding was that circulating immune cells play a central mediating role in this “gut–brain connection.” This result not only deepens our understanding of the pathogenesis of glioma but also provides new potential targets for future diagnostic methods and immunotherapy strategies.

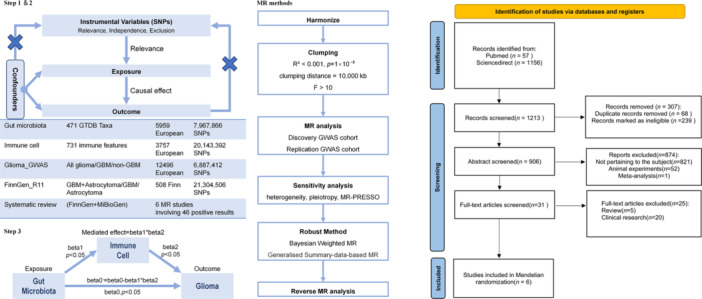

**Abbreviations**
AC: absolute cell count
BBB: blood‐brain barrier
cDC: classic dendritic cell
CI: confidence interval
FDR: false discovery rate
GBM: glioblastoma
GSMR: Summary‐data‐based Mendelian randomization
GTDB: Genomic Taxonomy Database
GWAS: genome‐wide association studies
HEIDI: Heterogeneity in Dependent Instruments
IV: instrumental variable
IVW: inverse variance weighted
MeSH: Medical Subject Headings
MIF: macrophage migration inhibitory factor
MR: Mendelian randomization
MR‐PRESSO: Mendelian Randomization Pleiotropy RESidual Sum and Outlier
NK: natural killer
OR: odds ratio
PRISMA: Preferred Reporting Items for Systematic Reviews and Meta‐Analyses
RC: relative cell count
ROBIS: Risk of Bias In Systematic Reviews
SCFA: short‐chain fatty acid
SE: standard error
SNP: single‐nucleotide polymorphism
STROBE‐MR: Strengthening the Reporting of Observational Studies in Epidemiology Using Mendelian Randomization
Treg: regulatory T cell
VEGFR2: vascular endothelial growth factor receptor 2
α‐GalCer: alpha‐galactosylceramide

## 1 Introduction

Gliomas are the most common malignant tumors of the central nervous system, accounting for up to 80% of all malignant primary brain tumors [1]. Among them, the 5‐year survival rate for GBM (WHO Grade 4 IDH wild type) is only 7.2%, with a near 100% recurrence rate [2]. Despite certain advancements in multidisciplinary treatments for gliomas, the efficacy remains limited, with an average survival time of just 14 months [2]. The heterogeneity of tumor cells and immunologic escape are two critical factors in the poor prognosis of gliomas.

Gut microbiota imbalance affects cancer progression and treatment response. It regulates local inflammation and immune responses, impacting whole‐body immunity. The discovery of the brain–gut axis provides compelling evidence for the relationship between gut microbiota and gliomas [3]. In a qualitative assessment and characterization of the gut microbiome in GBM patients, there was a significant increase in Proteobacteria at the phylum level and a decrease in Firmicutes in GBM patient feces compared to healthy fecal specimens. At the family level, the abundance of Enterobacteriaceae, Bacteroidaceae, and Lachnospiraceae was increased in GBM patients, while Veillonellaceae, Rikenellaceae, and Prevotellaceae were decreased. At the genus level, the abundance of Parasutterella, Escherichia‐Shigella, and Bacteroides was significantly increased in the GBM group, while the levels of Ruminococcus 2, Faecalibacterium, and Prevotella_9 were significantly reduced [4]. Ishaq et al. through high‐throughput sequencing, found that patients with GBM exhibit significantly increased microbial diversity. A notable decrease in Firmicutes and an increase in the families Coriobacteriaceae and Ruminococcaceae were observed, with significant reductions in the levels of Ruminococcus2 and Prevotella_9, demonstrating clear dysbiosis in GBM patients [4]. The regulatory role of the gut microbiota and immune cells is crucial for the occurrence and development of gliomas and has a potential impact on therapeutic efficacy [5]. Given the key role of the brain–gut axis in gliomas, our study aims to elucidate the causal associations between these complex traits and identify potential gut microbiota and targets for early diagnosis and clinical immunotherapy.

Genomics plays a significant role in understanding the occurrence and development of gliomas, and genome‐wide association studies (GWAS) have identified multiple genetic variants associated with glioma susceptibility, providing important clues for identifying potential etiology [6, 7]. MR is an innovative method for causal inference that uses specific genetic variants (single‐nucleotide polymorphisms [SNPs]) as objective and unique instrumental variables (IVs) to assess the potential causal effects between exposure factors and disease outcomes. Based on Mendelian inheritance laws, MR utilizes the random segregation and independent assortment of SNPs on gene loci, similar to randomized controlled trials, to reduce the interference of confounding factors and reverse causality. SNPs, as naturally occurring genetic markers, are not easily influenced by individual knowledge or external factors. SNP rs755622 in the promoter region of cytokine macrophage migration inhibitory factor (MIF) has been found to be associated with increased leukocyte infiltration in GBM [8]. The SNP rs2305948 on vascular endothelial growth factor receptor 2 (VEGFR2) has been found to be associated with glioma susceptibility in Asian populations [9].

Our study conducted a series of MR analyses to thoroughly investigate the impact of the gut microbiota on the risk of gliomas and their subtypes. We used multiple methods and systematic reviews to enhance the robustness of the results and further explored the complex role of the immune system. The study identified various gut microbiota with significant causal relationships with glioma subtypes and immune‐mediated effects, aiming to uncover the gut secrets of glioma susceptibility.

## 2 Materials and Methods

### 2.1 Study Design

**Figure 1:**
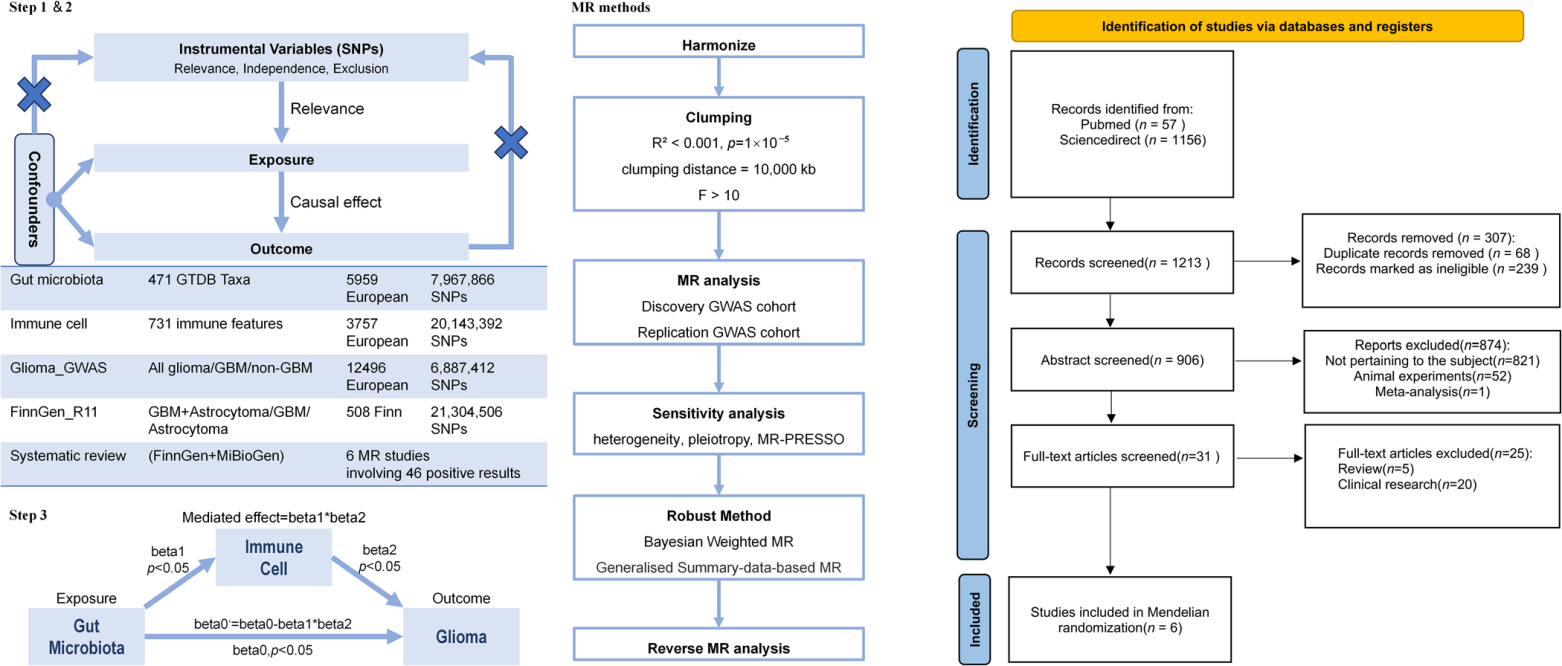
Workflow diagram.

This study was meticulously designed in accordance with the Strengthening the Reporting of Observational Studies in Epidemiology Using Mendelian Randomization (STROBE‐MR) guidelines [10]. The research was divided into three specific steps. In the first and second steps of the study, we utilized published GWAS, encompassing 471 gut microbiota Genomic Taxonomy Database (GTDB) traits, 731 immune cell traits, and three subtypes of glioma summary data. We selected appropriate SNPs as IVs for two‐sample MR to analyze bidirectional causal relationships between exposures and outcomes, and validated the results using external data and systematic reviews [11]. All data used in this study were derived from published GWAS summary statistics, and no individual‐level data were employed; therefore, ethical approval was not required for this study (data sources and workflow are depicted in Figure 1). In the third step of the study, we utilized the pairwise MR results between exposure‐mediation, mediation‐outcome, and exposure‐outcome to analyze the mediating effects of immune cells between gut microbiota and glioma outcomes, and calculated the effect size and proportion for each mediating factor.

### 2.2 Data Sources

For the exposure factor in this study, we utilized the most recent summary data on microbiota from Qin et al. which examined blood and fecal samples from 5959 European descendants in the FINRISK cohort, incorporating 7,967,866 SNPs as genetic characteristics. This study identified 471 distinct GTDB clusters, including 11 phyla, 19 classes, 24 orders, 62 families, 146 genera, and 209 species [12].

We considered the 731 immune cell traits reported by Orrù et al. as the mediating variables. This study data from 3757 European descendants, encompassing 20,143,392 mutation sites and covering various types of immune cells such as T cells, B cells, dendritic cells (DCs), monocytes, myeloid cells, natural killer (NK) cells, and regulatory T cells (Treg). The characteristics were categorized as follows: absolute cell counts (AC, *n* = 118), median fluorescence intensity reflecting surface antigen levels (MFI and SAL; *n* = 389), morphological parameters (MP; *n* = 32), and relative cell counts (RC; *n* = 192) [13].

The outcome data were derived from the largest glioma genetic susceptibility GWAS statistics to date (Glioma GWAS), which summarized data from eight independent European population samples. This included 12,496 cases (6191 classified as GBM and 5819 as non‐GBM tumors) and 18,190 controls that passed quality control, with GWAS SNP data for 6,887,412 SNPs [6]. All cases were histologically confirmed as gliomas (ICD‐O‐3), and the data were stratified based on malignancy. The external validation set for the outcome was derived from FinnGen_R11, using ICD‐O‐3 as the diagnostic criterion, encompassing 508 patients with GBM or Astrocytoma (GBM: 378, Astrocytoma: 130), and included 21,304,506 SNPs [14]. We matched this validation cohort with the three strata of Glioma_GWAS: Glioma_GWAS_All‐Glioma corresponded to C3_GBM_ASTROCYTOMA_EXALLC, Glioma_GWAS_GBM corresponded to C3_GBM_ASTROCYTOMA_EXALLC, and Glioma_GWAS_non‐GBM corresponded to C3_ASTROCYTOMA_EXALLC. Although this correspondence carries certain biases, given the consistency of the diagnostic criteria, we were able to derive objective results for GBM and Astrocytoma.

### 2.3 Selection of Instrumental Variables

To conduct a two‐sample MR study, the IVs must satisfy the following basic assumptions: (1) Relevance assumption: the selected SNPs are closely related to the exposure factor; (2) Independence assumption: the SNPs must be unrelated to potential confounding factors between exposure and outcome; (3) Exclusivity assumption: the SNP can only affect the outcome through the exposure factor. In two‐sample MR, we set the significance threshold for the IVs of the exposure factors (gut microbiota, immune cells, and glioma risk) to 1 × 10^−5^, a threshold widely accepted in multiple published MR studies to satisfy the independence assumption [12, 13, 15, 16]. As an exploratory study, the threshold (*p* < 1 × 10^−5^) is selected based on literature support, supplemented by linkage disequilibrium filtering (*R* ^2^ < 0.001, window 10,000 kb) and *F*‐statistic test (*F* > 10), which will ensure that IVs meet the three key assumptions of “relevance, independence, and exclusivity.” Additionally, Bayesian weighted Mendelian Randomization (BWMR) and generalized summary data Mendelian Randomization (GSMR) are employed to further correct potential pleiotropy and heterogeneity, thereby offsetting the risks arising from the relaxed threshold .

All IVs in this study underwent strict weak IV testing, with an *F*‐statistic > 10 used as the threshold for excluding weak IVs. This criterion was referenced from the guidelines of STROBE‐MR [10] and consensus from similar studies.

The calculation of *R* ^2^ adopted the standard formula (1) in genetic epidemiology:

(1) $$R^{2}=(2\beta^{2}\;p(1-p))/[2\beta^{2}p(1-p)+2\text{Np}(1-p)]$$

Calculation of the *F*‐statistic (The distribution of the *F*‐statistic: Figure S1) as the formula (2):

(2) $$F=(R^{2}(N-2))/(1-R^{2})$$

Gut microbiota traits‐SNP‐F statistic (GMs‐SNP‐F): mean 22.3, median 21.1, 5th to 95th percentile: 19.6–29.3; Immune cells‐SNP‐F statistic (IMCs‐SNP‐F): mean 34.4, median 21.7, 5th to 95th percentile: 19.7–63.6; Glioma Genome‐Wide Association Study‐SNP‐F statistic (GliomaGWAS‐SNP‐F): mean 45.1, median 23.3, 5th to 95th percentile: 19.8–187.0.

After screening with this threshold, the median number of SNPs for gut microbiota IVs was 18 (mean 19.5), 23 for immune cells (mean 25.6), and 48–57 for glioma. The F‐statistic of all IVs was > 10, indicating no weak IV bias (the IVs used in this study are presented in Tables S1–S3).

### 2.4 Two‐Sample MR Analysis

The two‐sample MR analysis was conducted using the Two Sample MR R package (version 0.6.6). We employed the inverse variance weighted (IVW) method as the primary approach to assess the correlation between exposure and outcome. The IVW method provides accurate and stable estimates when all IVs meet the three key assumptions. Results were presented as beta values with their standard errors (SEs) or odds ratios (OR) with their 95% confidence intervals (CIs), with *p* < 0.05 considered statistically significant. We accounted for the potential statistical errors due to multiple testing and used the false discovery rate (FDR) method for correction. As per previously reported studies, *p*‐values less than 0.05 but above the FDR‐adjusted threshold were considered to suggest a relationship [15]. Given that this was an exploratory study, the results we reported were not corrected for FDR. For results with *p* < 0.05 in the IVW MR analysis, we used additional supplementary MR analysis models as auxiliary methods to validate the significance of the findings, including the Wald ratio, Weighted Median, Weighted Mode, and MR‐Egger. Preliminary positive results from the MR were defined as those with *p* < 0.05 in the IVW model and the same direction of causal association as the MR–Egger method. The same analytical methods were applied to the external validation cohort.

### 2.5 Heterogeneity and Pleiotropy Tests

In this study, Cochran's *Q* test (based on IVW) method and MR–Egger model were used to evaluate the heterogeneity of IVs. A significant heterogeneity was indicated if *p* < 0.05. For horizontal pleiotropy, MR‐Egger intercept and MR‐PRESSO (Mendelian Randomization Pleiotropy RESidual Sum and Outlier) were applied for testing. If the intercept of MR–Egger regression significantly deviated from 0 (*p* < 0.05), it suggested the presence of horizontal pleiotropy, meaning that at least some SNPs affect the outcome through non‐exposure pathways. MR‐PRESSO analyzed the residual distribution of genetic variants (SNPs) via the Global Test, identified and removed outliers causing bias (*p* < 0.05), thereby obtaining more robust estimates of causal effects.

### 2.6 Sensitivity Analysis

The “leave‐one‐out” method was used for sensitivity analysis in this study. By sequentially excluding each SNP and re‐running IVW and MR‐Egger analyses, the changes in the combined effect size of the remaining SNPs were observed to verify the robustness of the causal effect estimates. If the direction of the results remained unchanged after excluding any SNP, it indicated that the conclusion was not driven by a single variant and had high reliability.

For the significant associations between gut microbiota characteristics (exposures) and glioma risk (outcome) identified in MR analyses, reverse causality tests were conducted to clarify the direction of the causal relationship.

### 2.7 BWMR and GSMR

To comprehensively address the challenges posed by weak IV bias and horizontal pleiotropy, we employed two advanced statistical frameworks: BWMR and GSMR [11, 17]. BWMR (version 0.1.1) integrates the variational expectation‐maximization (VEM) algorithm to estimate the posterior probability of causal effects while accounting for potential pleiotropic SNPs, thus providing more robust effect estimates. GSMR (gslmr2 version 1.1.1) can address two key limitations of traditional MR: first, it introduces the HEIDI (Heterogeneity in Dependent Instruments) test to exclude SNPs affected by pleiotropy through statistical testing (HEIDI *p* < 0.01); second, it directly models the genetic correlation between SNPs based on the generalized linear mixed model (GLMM), thereby avoiding potential impacts from linkage disequilibrium. Ultimately, only those results that successfully passed pleiotropy tests, heterogeneity tests, PRESSO tests, BWMR, and GSMR analyses were considered positive and were included in the final exploration of mediating effects.

### 2.8 Mediation Analysis

We selected potential immune mediators in the glioma‐gut microbiota pathway through the following steps, utilizing the IVW method as the analytical approach for the mediator effect size beta in this study. Initially, we identified immune mediators that demonstrated a significant causal effect with the positive exposure factors and calculated their effect sizes (beta1). Subsequently, we conducted a two‐sample MR for the aforementioned mediators against the outcome (Glioma_GWAS) and retained the effect sizes (beta2). To ensure the validity of the IVs, we checked for non‐redundancy among the IVs used to calculate beta1 and beta2 in the Two‐Step MR. In the third step, we extracted the causal effect size (beta0) of the positive exposure outcome on the outcome. Finally, we retained mediators that were logically consistent (if the total effect beta0 was positive, then both beta1 and beta2 should be either positive or negative; if the total effect beta0 was negative, then beta1 and beta2 should be one positive and one negative), and calculated the mediation effect size of the immune mediators using the “product of coefficients” method (beta1 × beta2) and the proportion of the effect size ([beta1 × beta2]/beta0) [18]. This design strictly follows the mediation analysis framework proposed by Sanderson [19], which ensures that the mediating variable is temporally situated between the exposure and the outcome by stepwise verifying the causal relationships between exposure and mediator, and between mediator and outcome. We have supplemented this point in the methodology section.

### 2.9 Systematic Literature Review Strategy

Adhering to the PRISMA guidelines, we conducted a systematic aggregation and review of MR studies examining the relationship between gut microbiota and glioma [20]. We identified relevant studies up to September 2024 using Medical Subject Headings (MeSH) indexing and keyword searches. We utilized disease‐related terms “glioma”, “Malignant Glioma”, and “glioblastoma” along with gut microbiota‐related terms “gut microbiota”, “intestinal microbiota”, “gut microbiome” and “intestinal microbiome” in PubMed and ScienceDirect to identify these studies. We included all MR studies concerning the gut microbiota and glioma. The studies encompassed the causal effects of GBM on gut microbiota and the intermediary factors associated with both. The following were excluded: (1) Various animal and cellular experiments, (2) Pure clinical trials, (3) Reviews and meta‐analyses, and (4) Lack of full‐text availability.

Our literature review was conducted entirely by two scholars in the field of glioma through manual identification. Initially, a preliminary screening was performed based on titles and abstracts, applying exclusion criteria to exclude any content that did not meet the predefined requirements. After the preliminary exclusion, a thorough and rigorous repeat review of the abstracts and full‐text articles was conducted. This detailed evaluation aimed to confirm whether each study met the specific inclusion criteria set by this systematic review. Any discrepancies in the assessment were resolved through collaboration between the two authors to ensure consensus on the final selection of studies included (the PRISMA flow diagram is shown in Figure 1). Then, we used the Risk of Bias In Systematic Reviews (ROBIS) tool to evaluate the bias of the systematic review. As the first rigorously validated methodological standard specifically designed to assess risk of bias in systematic reviews, this tool structures the evaluation of bias sources through four domains: Domain 1 (study eligibility criteria), Domain 2 (identification and selection of studies), Domain 3 (data collection and bias assessment) and Domain 4(synthesis and findings) [21]. The overall risk of bias for this study was rated as moderate (the ROBIS result is shown in Figure S2).

## 3 Results

### 3.1 Selection of Instrumental Variables

After applying the clump selection for IVs, the median number of SNPs for GMs was 18, with an average of 19.5. For immune cells, the median number of SNPs was 23, and the average was 25.6. The number of SNPs for Glioma_GWAS was as follows: All glioma had 53, GBM had 48, and non‐GBM had 57. All harmonized SNPs met the conditions of *r* ^2^ = 0.001, kb = 10000, and *p* < 0.05, ensuring that the *F*‐statistics were all greater than 10, with no weak IVs present (Tables S1–S3).

### 3.2 Causal Associations Between Gut Microbiota and Glioma Risk

**Figure 2:**
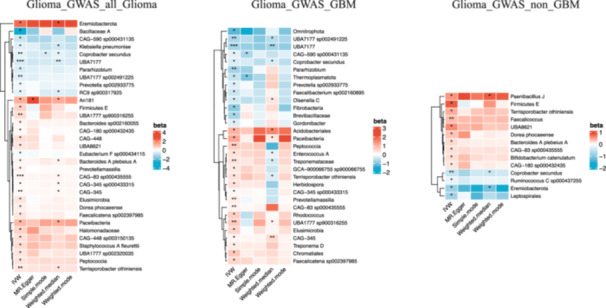
Significant causal relationship between intestinal flora and gliomas (IVW, *p* < 0.05), heatmap * denotes *p* < 0.05, ** denotes *p* < 0.01, and ***p < 0.001.

Among the 471 gut microbial populations included in the IVW analysis, 53 gut microbial populations were found to have significant causal associations with glioma (*p* < 0.05), encompassing 4 phyla, 4 classes, 3 orders, 5 families, 13 genera, and 24 species (as shown in Figure 2). When using Glioma_GWAS_all_glioma as the outcome, 23 microbial populations were positively associated with the onset of glioma, while 9 microbial populations were inversely related to the risk of glioma. Notably, CAG‐345 (OR = 1.116, *p* = 0.004), Terrisporobacter othiniensis (OR = 1.483, *p* = 0.004), and species from the family f_Oscillospiraceae, CAG‐83 sp000435555 (OR = 1.189, *p* < 0.001) and UBA1777 sp900316255 (OR = 1.708, *p* = 0.004), exhibited a more significant positive relationship with the development of glioma. In contrast, the species UBA7177 (OR = 0.560, *p* < 0.001,) and its genus UBA7177 sp002491225 (OR = 0.634, *p* = 0.008), along with *Coprobacter secundus* (OR = 0.677, *p* = 0.002), demonstrated a more significant protective effect against glioma. A total of 18 microbial taxa were found to promote GBM, while 13 taxa potentially protected against the GBM subtype. The GBM results further emphasized the significant positive association of fecal CAG‐83 sp000435555 (OR = 1.186, *p* = 0.004) and UBA1777 sp900316255 (OR = 1.938, *p* = 0.003) with GBM risk, in addition to Prevotellamassilia (OR = 1.269, *p* = 0.010), Treponemataceae (OR = 1.418, *p* = 0.010), and Peptococcia (OR = 1.916, *p* = 0.008), which were also significant risk factors. UBA7177 (OR = 0.463, *p* < 0.001) and its genus UBA7177 sp002491225 (OR = 0.504, *p* = 0.003), as well as Pararhizobium (OR = 0.464, *p* = 0.005), were found to have a protective effect against the development of GBM. For non‐GBM, 10 microbes showed a positive relationship, and only 4 microbes exhibited a protective effect. *C. secundus* (OR = 0.594, *p* = 0.001) and Faecalicoccus (OR = 1.413, *p* = 0.005) were identified as more significant protective and risk factors, respectively. In the MR analysis of GMs and GBM, Rhodococcus (*p* = 0.025) and UBA1777 sp900316255 (*p* = 0.048) did not pass the horizontal pleiotropy test, while all other results passed the heterogeneity test (*p* > 0.05), the PRESSO test, and the leave‐one‐out method test (Tables S4–S6).

To exclude the potential for reverse causal effects, we conducted a two‐sample MR analysis using the three types of gliomas as exposures and the significant results obtained from the IVW method as outcomes. We only identified one set of IVW significant causal effects, where GBM exerts a slight negative regulatory effect on the abundance of Elusimicrobia in stool (OR = 1.008, *p* = 0.028, *β* = 0.008) (Table S8).

### 3.3 Results of Validation Based on Systematic Review and External Datasets

We conducted a systematic review and identified a total of 1213 articles from the PubMed and ScienceDirect databases. After screening, we selected 6 MR studies that met our criteria [16, 22, 23, 24, 25, 26], which reported 46 positive results (*p* < 0.05). These results implicated 3 Phyla (Verrucomicrobia, Euryarchaeota, Cyanobacteria), 1 Order (Desulfovibrionales), 9 Families (Peptostreptococcaceae, Ruminococcaceae, Bacteroidaceae, Peptococcaceae, Streptococcaceae, Victivallaceae, Erysipelotrichaceae, Prevotellaceae, Rikenellaceae), 14 Genera (Adlercreutzia, catenibacterium, Coprobacter, Eubacterium brachy group, Anaerostipes, Faecalibacterium, Phascolarctobacterium, Streptococcus, Actinomyces, Bacteroides, Lactococcus, Eubacterium nodatum group, Ruminococcus gnarvus group, Lachnoclostridium, Selimonas), and 5 Species (Olsenella, Prevotella7, Lachnospiraceae UCG004, Ruminiclostridium6, Ruminococcaceae UCG002) (Table 1).

**Table 1:** Summary of positive results (*p* < 0.05) from six published Mendelian randomization (MR) studies identified by systematic review.

Year	DOI	Author	Exposure cases	GMs database	Outcome cases	Glioma database	Mean MR methods	Ivs thresholds	GMs	OR	95% CI	*p*	FDR
2024	10.1371/journal.pone.0304403	Chenzhi Cui	18,340	MiBioGen	3301	prot‐a‐1217(Gprp1)	IVW	1 × 10^−5^	Adlercreutzia	1.25	1.02–1.54	0.034	
									Catenibacterium	1.26	1.04–1.51	0.017	
									Coprobacter	1.17	1.01–1.36	0.041	
									Olsenella	0.86	0.75–0.99	0.048	
									Peptostreptococcaceae	0.67	0.57–0.86	0.001	
									Verrucomicrobia	1.27	1.01–1.57	0.043	
									Prevotella7	1.16	1.02–1.32	0.029	
									Euryarchaeota	1.13	1.00–1.30	0.049	
2024	10.3389/fgene.2023.1308263	Song Wang	18,340	MiBioGen	91	finn‐c3‐gbm	IVW	1 × 10^−5^	Peptostreptococcaceae	3.83	1.02–14.35	0.046	
									Eubacterium brachy group	2.85	1.16–7.01	0.023	
									Ruminococcaceae	0.04	0.01–0.19	< 0.001	a
									Anaerostipes	0.16	0.03–0.83	0.029	
									Faecalibacterium	0.16	0.04–0.65	0.011	
									Lachnospiraceae UCG004	0.2	0.04–0.96	0.045	
									Phascolarctobacterium	0.16	0.03–0.76	0.021	
									Prevotella7	0.3	0.13–0.68	0.004	
									Streptococcus	0.21	0.05–0.97	0.046	
2023	10.1186/s12864‐023‐09885‐2	Chuan Zeng	18,340	MiBioGen	91	finn‐c3‐gbm	IVW	1 × 10^−5^	Eubacterium brachy group	1.554	1.554–15.890	0.007	
									Eubacterium ruminantium group	3.673	1.087–12.411	0.036	
									Prevotella7	0.326	0.116–0.917	0.034	
									Peptostreptococcaceae	6.121	1.089–34.402	0.040	
									Ruminococcaceae	0.094	0.010–0.897	0.040	
2024	10.7150/jca.90149	Chao Ju	18,340	MiBioGen	162	finn‐c3‐gbm‐R8	IVW	1 × 10^−6^	Ruminococcaceae	0.094	0.021–0.417	0.019	
									Bacteroidaceae	12.003	1.793–80.32	0.010	
									Peptococcaceae	3.656	1.233–10.841	0.019	
									Eubacterium (brachy group)	4.431	1.529–12.842	0.006	
									Actinomyces	18.805	2.116–167.165	0.008	
									Bacteroides	12.003	1.794–80.320	0.010	
									Ruminiclostridium6	3.641	1.009–13.139	0.048	
2024	10.3389/fmicb.2024.1403316	Xuan Chen	18,340	MiBioGen	243	finn‐c3‐gbm‐R9	IVW	1 × 10^−5^	Streptococcaceae	0.36	0.15–0.89	0.026	
									Victivallaceae	1.95	1.21–3.13	0.005	
									Lactococcus	1.81	1.04–3.15	0.036	
									Desulfovibrionales	3.41	1.03–11.31	0.045	
									Cyanobacteria	0.45	0.22–0.89	0.021	
2024	10.3389/fneur.2024.1386885	Junqing Yan	18,340	MiBioGen	91	finn‐c3‐gbm	IVW	1 × 10^−5^	Erysipelotrichaceae	0.5624	0.3552–0.8903	0.014	
									Prevotellaceae	0.4242	0.2715–0.6626	< 0.001	
									Rikenellaceae	2.9845	2.1167–4.208	< 0.001	
									Victivallaceae	1.9541	1.2187–3.1332	0.005	
									Eubacterium nodatum group	0.6506	0.5124–0.8265	< 0.001	
									Ruminococcus gnarvus group	2.0719	1.614–2.6598	< 0.001	
									Lachnoclostridium	0.165	0.1161–0.2343	< 0.001	
									Lactococcus	1.8112	1.0399–3.1545	0.036	
									Ruminococcaceae UCG002	1.8835	1.1115–2.5497	0.014	
									Selimonas	1.8689	1.5679–2.2278	< 0.001	
									Desulfovibrionales	3.4129	1.0295–11.3141	0.045	
									Cyanobacteria	0.4463	0.2243–0.8878	0.022	

Abbreviations: FDR, false discovery rate; OR, odds ratios.

^a^ Causal relationship that remains significant after FDR correction.

**Figure 3:**
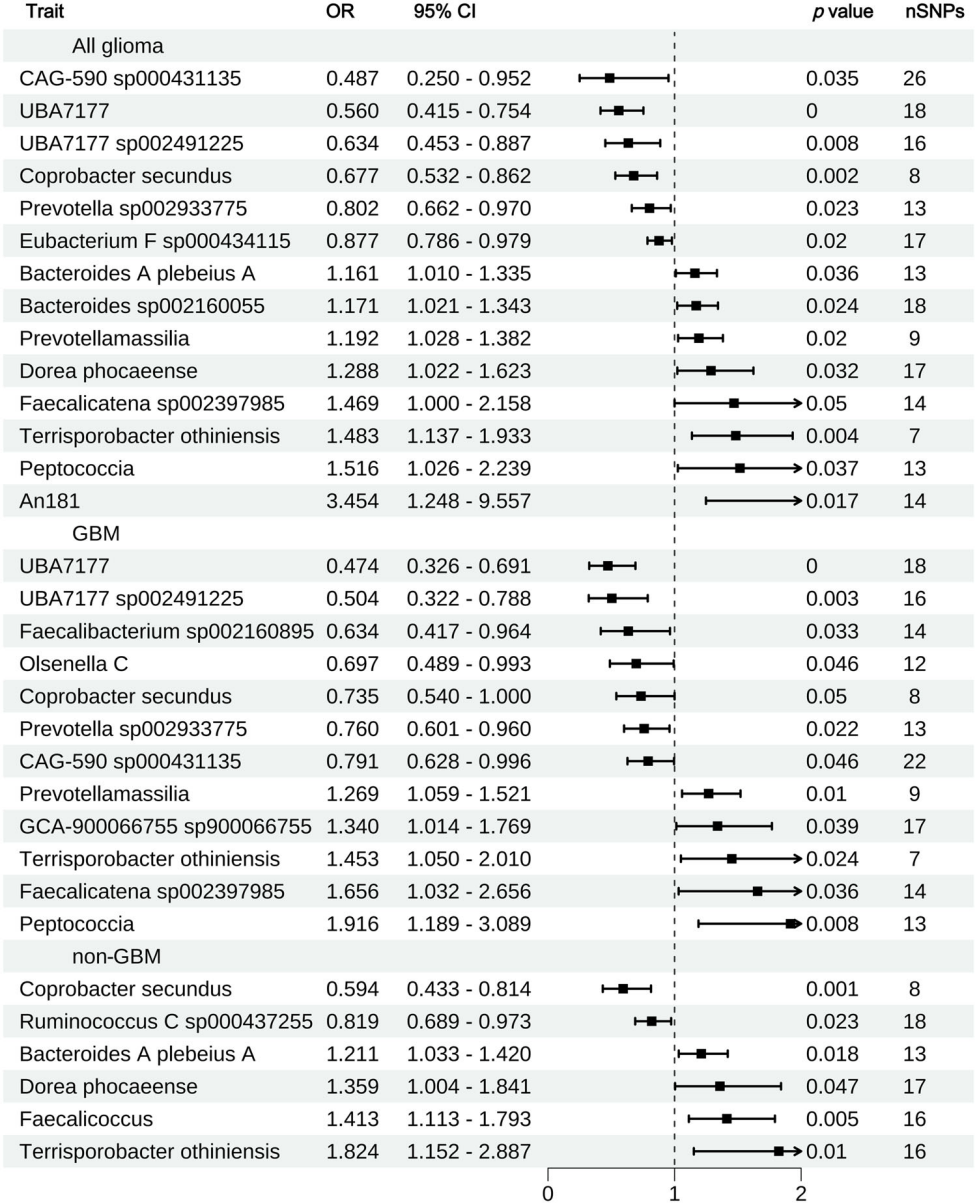
Causal relationships between gut microbiota and glioma supported by the literature review. nSNP, number of single‐nucleotide polymorphisms; OR, odds ratio.

Mapping these results to our three groups of MR findings, we identified 19 significant microbial entries (IVW, *p* < 0.05), confirming 6 Families previously reported (Bacteroidaceae, Erysipelotrichaceae, Lachnospiraceae, Ruminococcaceae, Peptostreptococcaceae, Peptococcaceae), 4 Genera (Prevotella, Coprobacter, Eubacterium, Faecalibacterium), and 1 Species (Olsenella) (Figure 3).

Using the same MR methods, we conducted a validation against the external data set Finn_R11. When using finn_r11_gbm_astro as the outcome, the positive result CAG‐83 sp000435555 (OR = 1.388, *p* = 0.042) and the negative result Eubacterium F sp000434115 (OR = 0.612, *p* = 0.019) were consistent with the results from Glioma_GWAS. When using finn_r11_gbm as the outcome, CAG‐590 sp000431135 (OR = 0.193, *p* = 0.044) was validated. When using finn_r11_astro as the outcome, only UBA8621 (OR = 29.387, *p* = 0.002) maintained the same directionality as the results from Glioma_GWAS. None of the above three results showed horizontal pleiotropy or heterogeneity (Tables S9 and S10).

**Figure 4:**
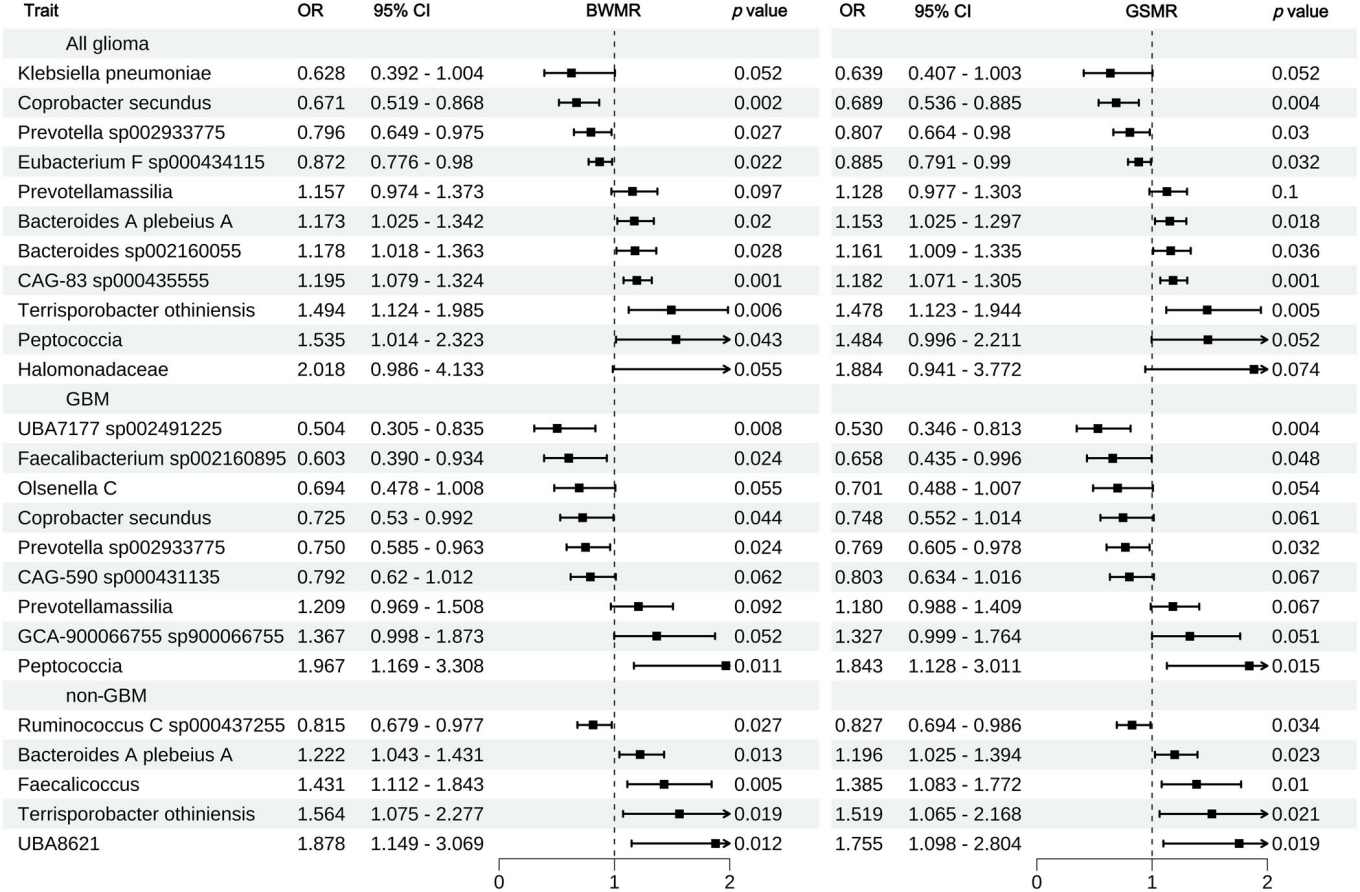
Causal relationships between gut microbiota and glioma strengthened by Bayesian weighted Mendelian randomization (BWMR) and generalized summary data Mendelian randomization (GSMR) enhanced analyses. nSNP, number of single‐nucleotide polymorphisms; OR, odds ratio.

To further avoid potential heterogeneity and pleiotropy, we performed additional BWMR and GSMR tests on the results that were validated by systematic review or external databases. We ultimately selected the microbial entries that met the significance criteria (*p* < 0.05) in both tests for further mediation analysis: seven, four, and five positive microbial entries for all glioma, GBM, and non‐GBM, respectively, passed this test and were considered potential microbial entries for mediation effect exploration (Figure 4, Table S7).

### 3.4 Causal Associations Between Immune Cell Phenotypes and Glioma Risk

**Figure 5:**
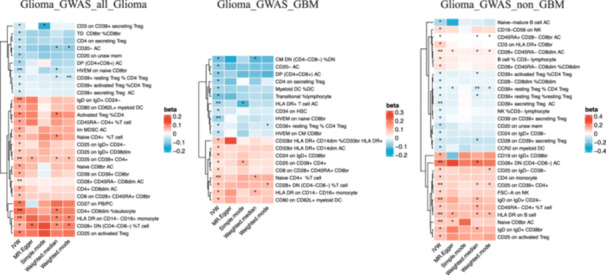
Significant causal relationship between plasma immune cells and gliomas (IVW, *p* < 0.05), * in heatmap indicates *p* < 0.05, ** indicates *p* < 0.01, and ***p < 0.001.

To identify potential immunological factors influencing glioma, we conducted a two‐sample MR analysis between immune cell phenotypes and three glioma outcomes, revealing that 58 immune cell phenotypes have a potential role in the occurrence of glioma. When Glioma_GWAS_all_glioma was used as the outcome, 6 B cell phenotypes, 1 classic dendritic cell (cDC) phenotype, 5 mature T cell phenotypes, 1 monocyte phenotype, 1 myeloid cell phenotype, 3 T/B/NK cell phenotypes, and 12 Treg phenotypes were found to have causal effects on various subtypes of glioma. For GBM, three B cell phenotypes, two cDC phenotypes, four mature T cell phenotypes, one monocyte phenotype, three myeloid cell phenotypes, one TBNK phenotype, and five Treg phenotypes were identified as potentially influencing GBM. For non‐GBM outcomes, in addition to 1 cDC and 2 mature T cell phenotypes, as many as 7 B cell and 7 T/B/NK phenotypes, as well as 13 Treg phenotypes, were found to influence non‐GBM outcomes. We subsequently excluded five immune cell phenotypes that did not meet the criteria for IV heterogeneity and pleiotropy, with the remaining results all passing sensitivity tests (Figure 5, Tables S11–S13).

### 3.5 Mediation Effect Exploration

**Figure 6:**
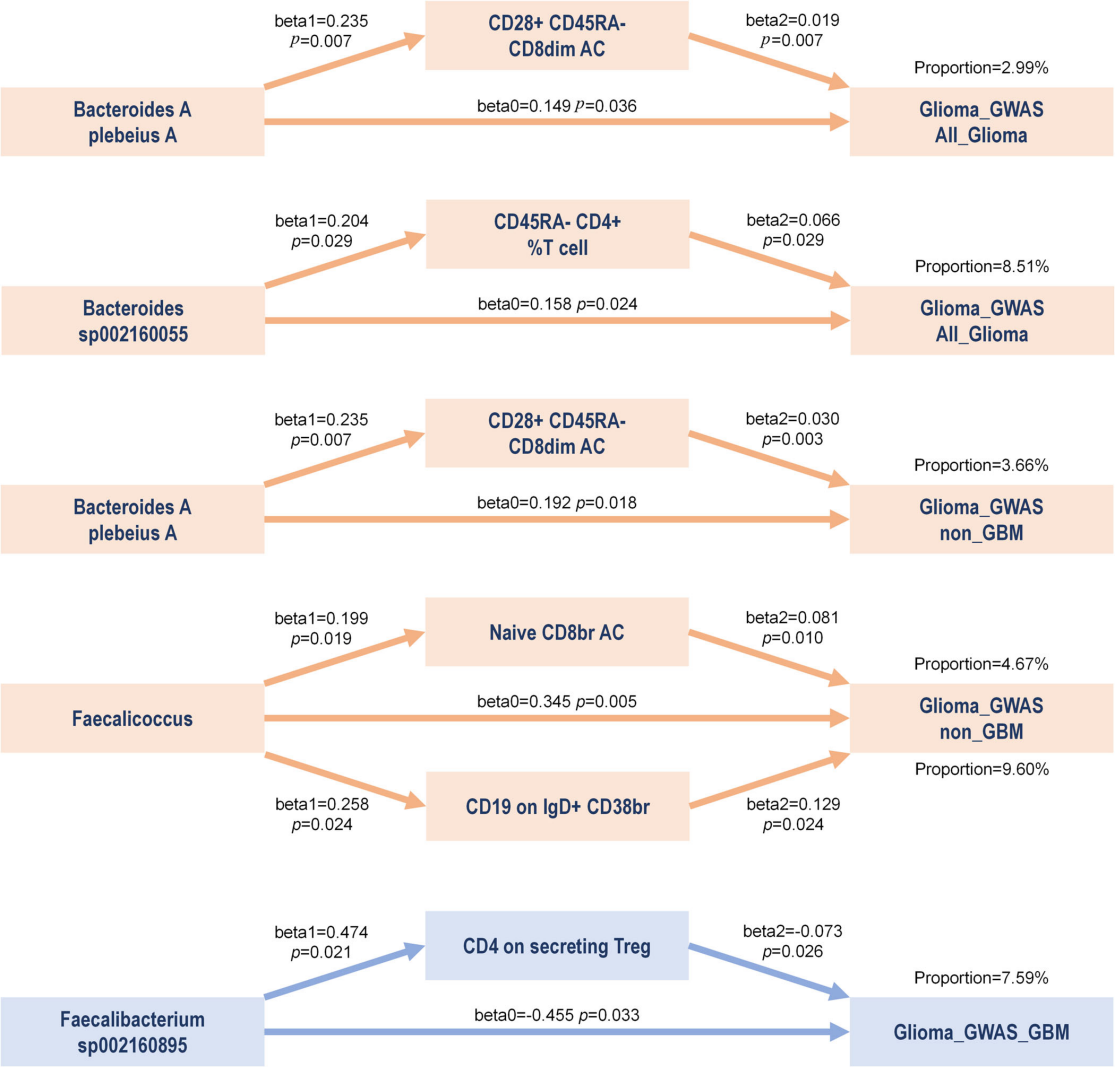
The mediation effect of immune traits between gut microbiota and glioma.

In this study, specific gut microbiota and immune cell phenotypes were found to have a causal relationship with glioma. Considering the potential immune‐mediated effects, we screened significant immune cell phenotypes and ultimately identified 14 gut microbiota‐immune cell‐glioma pathways (Tables S14 and S15). To ensure more reliable results, we conducted stringent sensitivity checks, and all pathways involving gut microbiota‐immune cell and immune cell‐glioma were analyzed using BWMR and GSMR, ultimately retaining four types of gut microbial communities and five immune cells. We calculated the mediation effect size and proportion for each significant pathway (Figure 6, Tables S16–S18). The results indicated that CD28^+^CD45RA^−^ CD8dim AC mediated the risk of Bacteroides A plebeius A on glioma in both all_glioma and non_GBM, with mediation effect proportions of 2.99% and 3.66%, respectively. CD45RA^−^CD4^+^ %T cell mediated the risk of Bacteroides sp002160055 on all_glioma, with a proportion of 8.51%. Naive CD8br AC and CD19 on IgD+ CD38br both participated in mediating the causal relationship between Faecalicoccus and non‐GBM (4.67% and 9.6%). We identified a protective factor for GBM, Faecalibacterium sp002160895, which also has the potential to increase the expression of CD4 on secreting Treg (beta1 = 0.474, *p* = 0.021). Moreover, CD4 on secreting Treg also had a negative effect on the development of GBM (beta2 = −0.073, *p* = 0.026), accounting for 7.59% of the protective effect in the gut microbiota‐immune cell‐glioma pathway.

## 4 Discussion

The discovery of the brain–gut axis has progressively unveiled the relationship between gut microbiota and neurological diseases, including glioma [4]. Our study, employing a comprehensive bidirectional MR framework combined with a systematic review, provides robust genetic evidence supporting a causal role of specific gut microbial taxa in glioma susceptibility. Importantly, we delineate the critical mediating effects of circulating immune cells within this relationship, identifying key gut microbiota‐immune‐glioma pathways. These findings emphasize the brain–gut–immune axis as a key modulator of glioma pathogenesis and reveal potential targets for biomarker discovery and therapeutic intervention.

We report for the first time multiple gut microbial communities, primarily within the phyla Firmicutes and Bacteroidota, with potential causal links to glioma development. Previous MR studies have shown considerable heterogeneity, with the majority suffering from low statistical power due to small outcome sample sizes. Our large sample size, multiple replications, and enhanced analyses further substantiate the reliability of our results. Our results validate some of the previously published MR conclusions, but there are also some discrepancies.

The findings regarding Bacteroides, Coprobacter, and Ruminococcaceae align with previous conclusions, whereas opposing results were observed for Eubacterium and Prevotella. Bacteroides, with increasing abundance in gut microbiota, raises the risk of GBM multiforme. This bacterium produces short‐chain fatty acids (SCFAs) that regulate the growth and metabolism of gliomas by affecting immune responses, angiogenesis, and epigenetic modifications [25]. *B. fragilis* toxin promotes chronic intestinal inflammation, activates Tregs via the STAT3 pathway, and enhances Th17 activity. Pathogenic Th17 cells produce IL‐17 and IL‐6, which contribute to tissue damage, inflammation, and ultimately oncogenesis by promoting tumor cell survival, proliferation, angiogenesis, and metastasis [27]. Our mediation analysis revealed that the risk effect of Bacteroides A plebeius A on glioma was mediated by CD28^+^CD45RA^−^CD8dim Treg cells, suggesting a pathway where microbiota influences T‐cell regulation, potentially fostering a permissive tumor microenvironment.

Conversely, the protective effect of Faecalibacterium sp002160895 was mediated by CD4 expression on secreting Treg cells. This aligns with preclinical studies showing that Faecalibacterium prausnitzii, a congener, enhances antitumor immune effects of immune checkpoint inhibitors (ICIs) by promoting CD8^+^T‐cell infiltration and cytokine production, such as interferon‐γ (IFN‐γ) and tumor necrosis factor‐α (TNF‐α) [28]. Faecalibacterium. prausnitzii stabilizes Tregs via SCFA‐mediated inhibition of histone deacetylase (HDAC) [29]_._ The dose‐dependent nature of host‐microbiota interactions suggests that the balance between pro‐tumor and antitumor taxa is critical, potentially explaining why a high abundance of certain Bacteroides may disrupt the Th17/Treg balance towards inflammation and tumor progression [30]. Animal experiments related to Coprobacter have shown that this bacterium enhances the efficacy of immune checkpoint blockade (ICB) therapy, modulating CTLA‐4 or PD‐L1, pointing to a new direction for future cancer treatments [22, 31].

The role of Eubacterium in health is still a matter of considerable debate. Our findings suggest a potential protective effect of certain species, which contrasts with the pro‐tumor role proposed by Wang et al., where E. rectale was identified as a potential “driver” for colorectal cancer (CRC) initiation by fostering an inflammatory microenvironment [32]. This discrepancy likely originates from the considerable functional heterogeneity within this phylogenetically diverse genus [33]. For instance, while Eubacterium produces acetate—a bioenergetic substrate for GBM that could theoretically support tumor proliferation [34]—it also produces butyrate, which can inhibit HDAC and induce cell cycle arrest. Furthermore, antigenic peptides derived from Eubacterium have been shown to activate tumor‐infiltrating lymphocytes (TILs) [35], suggesting that specific species, such as the Eubacterium F sp000434115 identified in our study, might confer protection by enhancing antitumor immunity. This underscores the critical influence of species‐ or strain‐level differences on its functional outcome in glioma pathogenesis.

A notable finding was the protective association of Prevotella with glioma, which contrasts with some previous studies reporting its pro‐inflammatory, cancer‐promoting role [22, 36]. This discrepancy highlights the significant functional heterogeneity at the strain level within a genus. For example, Prevotella copri has been reported to produce α‐GalCer in mouse GBM tissue, stimulating γδT cells or invariant natural killer T (iNKT) cells to exert direct or indirect anticancer effects [37], while other strains like Prevotella 7 may promote inflammation via Toll‐like receptor 2 (TLR2)/interleukin‐23 (IL‐23) signaling [36]. The ultimate effect likely depends on a triad of factors: the specific bacterial strain, the host's genetic background, and environmental influences like diet. The Prevotella 7 strain belongs to a different subtype from the bacteria we are studying. The functional differences at the strain level are the core reason for the contradiction in the conclusion. Besides, the host genotype may significantly influence the microbial‐host interaction. People with acquired mutations in the TLR2 function may be more sensitive to the pro‐inflammatory effects of Prevotella 7, while the genetic polymorphism of the gene encoding the α‐GalCer synthase may determine whether an individual can effectively utilize the anticancer function of this bacterium. Furthermore, a high‐fiber diet can promote the production of SCFAs by Provorotra bacteria, enhancing their anticancer effects; while a high‐fat diet may induce the bacteria to shift to an inflammatory metabolic phenotype. Provorotra bacteria have an ecological niche competition with the Bacteroidetes phylum, and the abundance of the latter may affect the functional expression of the former. In the future, it is necessary to further analyze the triadic interaction among the bacterial strain, the host, and the environment by integrating multi‐omics data (such as whole‐genome sequencing and metabolomics).

Our results validate the protective role of Ruminococcaceae family bacteria, consistent with findings from several prior MR studies and observations in other cancers, such as melanoma and liver cancer [38, 39]. This family's association with improved response to anti‐PD‐1 immunotherapy in melanoma suggests a conserved mechanism of enhancing tumor immune surveillance, possibly through metabolic regulation of the tumor microenvironment [29, 38]. The implication is that certain gut microbial features might predict response to immunotherapy not only in peripheral cancers but also in central nervous system malignancies, especially considering the compromised blood‐brain barrier in glioma allows for greater immune cell migration [40, 41].

Our findings lend robust support to the established concept that gut microbiota dysbiosis can contribute to glioma pathogenesis through immune dysregulation [35]. The gut microbiota exerts systemic immunomodulatory effects by shaping the development and function of the intestinal immune system, with approximately 70% of the body's immune cells residing in the gut‐associated lymphoid tissue [42, 43, 44]. During the development of glioma, the blood–brain barrier (BBB) is compromised, allowing various immune cells to infiltrate the tumor surroundings, providing a theoretical basis for the application of gut microbiota and their derived antigenic peptides in the treatment of central nervous system tumors [40, 41].

The identified microbiota‐immune pathways hold substantial translational promise. For diagnosis, a composite model integrating fecal abundance of specific microbes (e.g., Faecalicoccus) with plasma levels of specific immune cells (e.g., CD19^+^IgD^+^CD38bright B cells) could facilitate noninvasive risk stratification for glioma, potentially identifying high‐risk individuals before radiological detection. Therapeutically, modulating the gut microbiome presents a viable strategy. Supplementation with protective taxa like Faecalibacterium sp002160895 could potentially sensitize “immune‐cold” gliomas to immune checkpoint inhibitors by enhancing Treg‐mediated antitumor responses. Furthermore, engineered probiotics expressing immunomodulators (e.g., IL‐12) or metabolites that counteract oncogenic bacterial products represent a frontier for next‐generation microbiome‐based therapies. Fecal microbiota transplantation (FMT) enriched with protective consortia (e.g., Ruminococcaceae) could also be explored in adjuvant settings.

Future validation necessitates a multi‐tiered approach: a prospective cohort tracking microbiome‐immune dynamics; humanized mouse models testing candidate strains (Faecalicoccus vs. Faecalibacterium); and Phase I trials of FMT capsules enriched with protective taxa. This systematic approach will accelerate clinical translation of microbiome‐based therapies.

The modest mediation proportions (2.99%–9.6%) may arise from direct microbiota effects through metabolites; synergistic effect of multiple immune mediators (such as B cells and T cell subsets); and limitations in capturing functional immune states. Future studies should integrate single‐cell technologies and functional validation.

This study has several limitations. First, to obtain sufficient IVs, we set the SNP significance threshold at 1 × 10^−5^. Although we conducted comprehensive sensitivity analyses, including leave‐one‐out, horizontal pleiotropy, and MR‐PRESSO tests to ensure robustness, potential residual bias may persist. Second, the external validation data set (FinnGen_R11) had a limited sample size (508 cases total), and the classification differences between astrocytoma in the validation set and non‐GBM in Glioma_GWAS resulted in fewer validation outcomes. To address these issues, we performed a systematic literature review of previous MR studies to further supplement and validate our conclusions. Our findings demonstrate strong persuasiveness for GBM outcomes, warranting future investigations into gut microbiota in other glioma subtypes.

Furthermore, methodological differences may introduce bias: FINRISK represents a general population cohort, while the Glioma GWAS employs a case–control design. Glioma treatments and progression may alter gut microbiota composition, differing from healthy baseline levels. Although both datasets are European‐derived, genetic and dietary variations between Finnish and other European populations may limit generalizability to other ethnic groups.

To address limited ethnic diversity, we plan to establish multiethnic cohorts to validate conserved pathways; develop ethnic‐specific genomic databases; and conduct cross‐population functional studies. These initiatives will enhance the generalizability and translational potential of our findings.

## 5 Conclusion

In summary, this study thoroughly explored the causal relationship between gut microbiota and glioma and analyzed the mediating role of the immune system. Through replicated, two‐sample, bidirectional MR studies and PRISMA systematic reviews, we found that gut microbiota imbalance is closely related to the occurrence of glioma and identified multiple gut microbial communities and immune‐mediated effects with significant causal relationships to glioma subtypes. Our findings emphasize the role of the brain–gut axis in glioma and provide potential targets for early diagnosis and clinical immunotherapy.

## Author Contributions

**Jiachen Wang:** data curation (equal), formal analysis (equal), methodology (equal), writing – original draft (equal), writing – review and editing (equal). **Yilin Zhang:** data curation (equal), formal analysis (equal), methodology (equal), writing – original draft (equal), writing – review and editing (equal). **Zhuang Kang:** writing – review and editing (equal). **Shenglan Li:** funding acquisition (equal), writing – review and editing (equal). **Rong Zhang:** writing – review and editing (equal). **Mengqian Huang:** writing – review and editing (equal). **Chengzhuo Wang:** methodology (equal), writing – review and editing (equal). **Yuxiang Fan:** methodology (equal), writing – review and editing (equal). **Xinrui Liu:** writing – review and editing (equal). **Yuxiao Chen:** writing – review and editing (equal). **Tingrui Han:** writing – review and editing (equal). **Yuji Wang:** conceptualization (equal), project administration (equal). **Wenbin Li:** conceptualization (equal), funding acquisition (equal), project administration (equal).

## Ethics Statement

The authors have nothing to report.

## Consent

The authors have nothing to report.

## Conflicts of Interest

Professor Wenbin Li is a member of the *Cancer Innovation* Editorial Board. To minimize bias, he was excluded from all editorial decision‐making related to the acceptance of this article for publication. The other authors declare no conflicts of interest.

## Supporting information

STROBE‐MR checklist of recommended items to address in reports of Mendelian randomization studies.

figureS1.

figureS2.

Table S.

## Data Availability

The authors declare that the data supporting the findings of this study are available within the article and its Supporting Information S1 files. Other data comes from public databases.
