# Supplementary material for: Bidirectional Causal Effect Between Gut Microbiota and Glioma Risk: A Systematic Review‐Based Mendelian Randomization and Immune‐Mediated Effect Analysis
Source: Cancer Innov. 2025 Dec 9;4(6):e70039. doi: 10.1002/cai2.70039 (PMC12689236; doi:10.1002/cai2.70039)
Supplement: Supplementary file 1 — STROBE‐MR checklist of recommended items to address in reports of Mendelian randomization studies. [file CAI2-4-e70039-s001.docx]

**­­STROBE-MR checklist of recommended items to address in reports of Mendelian randomization studies**^1^ ^2^

| **Item No.** | **Section** | **Checklist item** | **Page No.** | **Relevant text from manuscript** |
| --- | --- | --- | --- | --- |
| 1 | **TITLE and ABSTRACT** | Indicate Mendelian randomization (MR) as the study’s design in the title and/or the abstract if that is a main purpose of the study | 1,3 | Immunological Dissection of the Glioma-Gut Axis: A Replicated Mendelian Randomization Study and Mediating Analysis; Glioma is the most common…diagnosis and treatment of glioma. |
|  | **INTRODUCTION** |  |  |  |
| 2 | **Background** | Explain the scientific background and rationale for the reported study. What is the exposure? Is a potential causal relationship between exposure and outcome plausible? Justify why MR is a helpful method to address the study question | 4,5 | Gliomas are the most common…; The imbalance of the gut microbiota…relationship between gut microbiota and gliomas; Mendelian randomization (MR) is an innovative method…glioma susceptibility in Asian populations. |
| 3 | **Objectives** | State specific objectives clearly, including pre-specified causal hypotheses (if any). State that MR is a method that, under specific assumptions, intends to estimate causal effects | 5 | Our study conducted series of MR analysis to…uncover the gut secrets of glioma susceptibility. |
|  | **METHODS** |  |  |  |
| 4 | **Study design and data sources** | Present key elements of the study design early in the article. Consider including a table listing sources of data for all phases of the study. For each data source contributing to the analysis, describe the following: |  |  |
|  | a) | Setting: Describe the study design and the underlying population, if possible. Describe the setting, locations, and relevant dates, including periods of recruitment, exposure, follow-up, and data collection, when available. | 5,6 | For the exposure factor in this study, we utilized the most recent summary data on microbiota from Qin et al…to C3_ASTROCYTOMA_EXALLC. |
|  | b) | Participants: Give the eligibility criteria, and the sources and methods of selection of participants. Report the sample size, and whether any power or sample size calculations were carried out prior to the main analysis | 5,6 | from 5,959 European descendants in the FINRISK cohort; from 3,757 European descendants; This included 12,496 cases… |
|  | c) | Describe measurement, quality control and selection of genetic variants | 6 | We used clump to remove linkage disequilibrium among IVs (R² < 0.001…frequencies. (The IVs used in this study are presented in Table S1, S2, and S3) |
|  | d) | For each exposure, outcome, and other relevant variables, describe methods of assessment and diagnostic criteria for diseases | 5,6 | This study identified 471 distinct Genomic Taxonomy Database (GTDB) clusters…using ICD-O-3 as the diagnostic criterion…or GBM and Astrocytoma. |
|  | e) | Provide details of ethics committee approval and participant informed consent, if relevant | 5 | All data used in this study were derived from published …ethical approval was not required for this research. |
| 5 | **Assumptions** | Explicitly state the three core IV assumptions for the main analysis (relevance, independence and exclusion restriction) as well assumptions for any additional or sensitivity analysis | 6 | To conduct a two-sample MR study, the instrumental variables (IVs) must satisfy the following basic assumptions:…can only affect the outcome through the exposure factor. |
| 6 | **Statistical methods: main analysis** | Describe statistical methods and statistics used |  |  |
|  | a) | Describe how quantitative variables were handled in the analyses (i.e., scale, units, model) |  | NA |
|  | b) | Describe how genetic variants were handled in the analyses and, if applicable, how their weights were selected | 6,7 | In two-sample MR, we set the significance threshold for the IVs of the exposure factors…The same analytical methods were applied to the external validation cohort. |
|  | c) | Describe the MR estimator (e.g. two-stage least squares, Wald ratio) and related statistics. Detail the included covariates and, in case of two-sample MR, whether the same covariate set was used for adjustment in the two samples | 7 | We employed the inverse variance weighted (IVW) method as the primary approach…The same analytical methods were applied to the external validation cohort. |
|  | d) | Explain how missing data were addressed |  | NA |
|  | e) | If applicable, indicate how multiple testing was addressed | 7 | We accounted for the potential statistical errors due to multiple testing…suggest a relationship |
| 7 | **Assessment of assumptions** | Describe any methods or prior knowledge used to assess the assumptions or justify their validity | 6 | We calculated the F-statistic to measure the strength of the IVs…SNPs or SNPs with incompatible effect allele frequencies. |
| 8 | **Sensitivity analyses and additional analyses** | Describe any sensitivity analyses or additional analyses performed (e.g. comparison of effect estimates from different approaches, independent replication, bias analytic techniques, validation of instruments, simulations) | 7 | Heterogeneity among individual genetic variants in the IVW and MR-Egger methods was assessed using Cochrane’s Q test…bidirectional causality. |
| 9 | **Software and pre-registration** |  |  |  |
|  | a) | Name statistical software and package(s), including version and settings used | 7 | TwoSampleMR R package (version 0.6.6); BWMR, version 0.1.1; GSMR, gslmr2 version 1.1.1; MR-PRESSO |
|  | b) | State whether the study protocol and details were pre-registered (as well as when and where) | 5 | The protocol of the study has been registered on OSF (10.17605/OSF.IO/TP672) , and there is no discrepancy between the registered protocol and the current research. |
|  | **RESULTS** |  |  |  |
| 10 | **Descriptive data** |  |  |  |
|  | a) | Report the numbers of individuals at each stage of included studies and reasons for exclusion. Consider use of a flow diagram | 5,6 | from 5,959 European descendants in the FINRISK cohort; from 3,757 European descendants; This included 12,496 cases…; The workflow chart is shown in Figure 1. |
|  | b) | Report summary statistics for phenotypic exposure(s), outcome(s), and other relevant variables (e.g. means, SDs, proportions) | 5,6 | 5,959 European descendants in the FINRISK cohort, incorporating 7,967,866 SNPs …; Summary statistics information can be found in Figure 1 and Supplementary Tables 1, 2, and 3. |
|  | c) | If the data sources include meta-analyses of previous studies, provide the assessments of heterogeneity across these studies | NA | The heterogeneity of the meta-analysis used is reported in the supplementary file(The source, size, location, and heterogeneity of each SNP are reported in the supplementary tables) |
|  | d) | For two-sample MR:  i.  Provide justification of the similarity of the genetic variant-exposure associations between the exposure and outcome samples  ii.  Provide information on the number of individuals who overlap between the exposure and outcome studies | 5,6 | The samples used in the study were all from individuals of European descent, and there were no instances of sample overlap that needed to be reported. |
| 11 | **Main results** |  |  |  |
|  | a) | Report the associations between genetic variant and exposure, and between genetic variant and outcome, preferably on an interpretable scale | 9 | The association between the instrumental variable and the exposure and outcome is reported in the supplementary materials. |
|  | b) | Report MR estimates of the relationship between exposure and outcome, and the measures of uncertainty from the MR analysis, on an interpretable scale, such as odds ratio or relative risk per SD difference | 9 | Among the 471 gut microbial populations included in the IVW analysis, 53 gut microbial populations were found to have significant causal associations with glioma (p<0.05)…(Figure 2, Table S4,5, supplementary material). |
|  | c) | If relevant, consider translating estimates of relative risk into absolute risk for a meaningful time period |  | NA |
|  | d) | Consider plots to visualize results (e.g. forest plot, scatterplot of associations between genetic variants and outcome versus between genetic variants and exposure) | 9,10 | Figure 2. All forest plots and scatterplots, are provided in the supplementary materials. |
| 12 | **Assessment of assumptions** |  |  |  |
|  | a) | Report the assessment of the validity of the assumptions | 9,10 | Multiple assessment of validity are reported throughout the text, including the F-statistic for the model and the Q-statistic, which can be found in the supplementary materials. |
|  | b) | Report any additional statistics (e.g., assessments of heterogeneity across genetic variants, such as *I^2^*, Q statistic or E-value) |  | NA |
| 13 | **Sensitivity analyses and additional analyses** |  |  |  |
|  | a) | Report any sensitivity analyses to assess the robustness of the main results to violations of the assumptions | 9,10 | Figures 2, 3, and 4 accurately report the results of various MR (Mendelian Randomization) methods used to assess the robustness of the findings,all results are available in the supplementary tables.. |
|  | b) | Report results from other sensitivity analyses or additional analyses | 9,10 | We reported horizontal pleiotropy and heterogeneity in our results, and all results are available in the supplementary tables. |
|  | c) | Report any assessment of direction of causal relationship (e.g., bidirectional MR) | 9,10 | To exclude the potential for reverse causal effects, we conducted a two-sample MR analysis using the three types of glioma as exposures… |
|  | d) | When relevant, report and compare with estimates from non-MR analyses | 10,11,12 | We employed a systematic literature review methodology to thoroughly assess our results(Table 1). |
|  | e) | Consider additional plots to visualize results (e.g., leave-one-out analyses) | 9~11 | Figure 2~5. All forest plots and scatterplots, are provided in the supplementary materials. |
|  | **DISCUSSION** |  |  |  |
| 14 | **Key results** | Summarize key results with reference to study objectives | 11,12,13 | We report for the first-time multiple gut microbial communities with potential causal relationships to the development of glioma, including Bacteroides A plebeius A, Bacteroides sp002160055… |
| 15 | **Limitations** | Discuss limitations of the study, taking into account the validity of the IV assumptions, other sources of potential bias, and imprecision. Discuss both direction and magnitude of any potential bias and any efforts to address them | 14,15 | Our study may have the following limitations: to comprehensively… |
| 16 | **Interpretation** |  |  |  |
|  | a) | Meaning: Give a cautious overall interpretation of results in the context of their limitations and in comparison with other studies | 12,13,14 | Previous MR studies have shown considerable heterogeneity, with the majority suffering from low statistical power due to small outcome sample sizes. Our large sample size, multiple… |
|  | b) | Mechanism: Discuss underlying biological mechanisms that could drive a potential causal relationship between the investigated exposure and the outcome, and whether the gene-environment equivalence assumption is reasonable. Use causal language carefully, clarifying that IV estimates may provide causal effects only under certain assumptions | 12,13,14,15 | We have elaborated on the potential mechanisms in detail in the discussion section. |
|  | c) | Clinical relevance: Discuss whether the results have clinical or public policy relevance, and to what extent they inform effect sizes of possible interventions | 14,15 | We have expounded on the clinical relevance in our discussion section. |
| 17 | **Generalizability** | Discuss the generalizability of the study results (a) to other populations, (b) across other exposure periods/timings, and (c) across other levels of exposure | 14,15 | Additionally, our study population consists entirely of individuals…and may not be fully applicable to other ethnic groups. |
|  | **OTHER INFORMATION** |  |  |  |
| 18 | **Funding** | Describe sources of funding and the role of funders in the present study and, if applicable, sources of funding for the databases and original study or studies on which the present study is based | 16 | This work is supported by the Talent Introduction... |
| 19 | **Data and data sharing** | Provide the data used to perform all analyses or report where and how the data can be accessed, and reference these sources in the article. Provide the statistical code needed to reproduce the results in the article, or report whether the code is publicly accessible and if so, where | 15 | The authors declare that the data supporting the findings of this study are available within the ... |
| 20 | **Conflicts of Interest** | All authors should declare all potential conflicts of interest | 15 | The authors declare no competing interests or... |

This checklist is copyrighted by the Equator Network under the Creative Commons Attribution 3.0 Unported (CC BY 3.0) license.

1. Skrivankova VW, Richmond RC, Woolf BAR, Yarmolinsky J, Davies NM, Swanson SA, et al. Strengthening the Reporting of Observational Studies in Epidemiology using Mendelian Randomization (STROBE-MR) Statement. JAMA. 2021;under review.

2. Skrivankova VW, Richmond RC, Woolf BAR, Davies NM, Swanson SA, VanderWeele TJ, et al. Strengthening the Reporting of Observational Studies in Epidemiology using Mendelian Randomisation (STROBE-MR): Explanation and Elaboration. BMJ. 2021;375:n2233.
