# Supplementary material for: Bidirectional Causal Effect Between Gut Microbiota and Glioma Risk: A Systematic Review‐Based Mendelian Randomization and Immune‐Mediated Effect Analysis
Source: Cancer Innov. 2025 Dec 9;4(6):e70039. doi: 10.1002/cai2.70039 (PMC12689236; doi:10.1002/cai2.70039)
Supplement: Supplementary file 2 — figureS1. [file CAI2-4-e70039-s003.pdf]

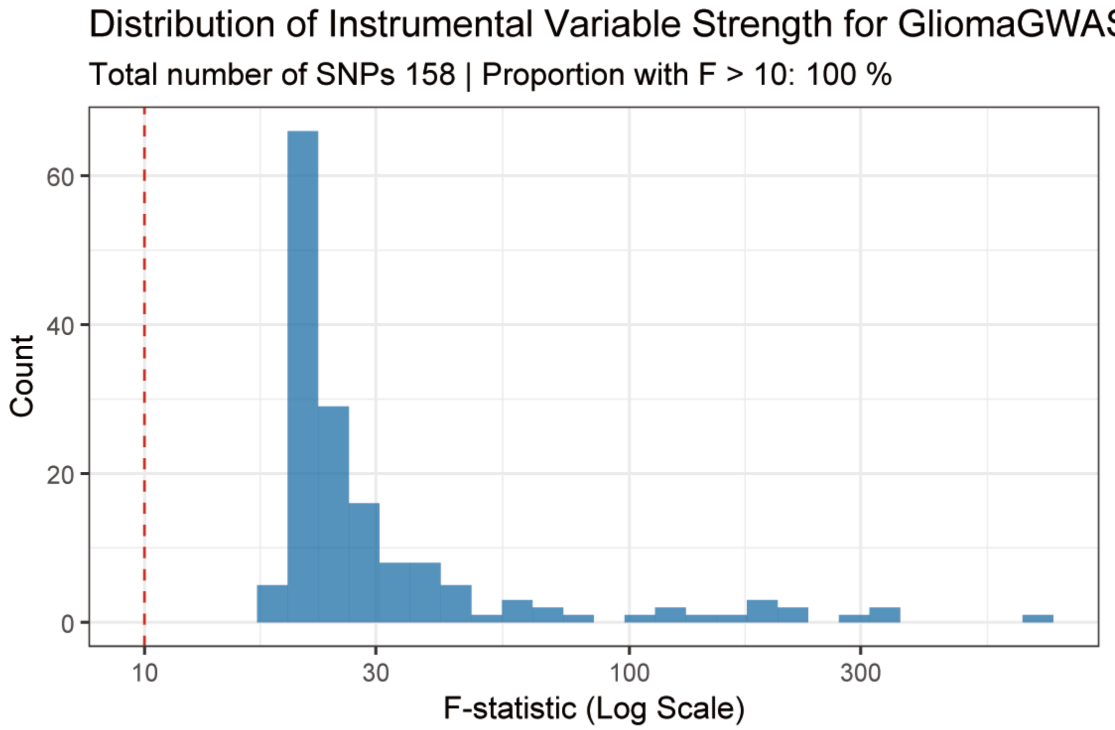
Distribution of Instrumental Variable Strength for GliomaGWA
Total number of SNPs 158 | Proportion with F > 10: 100 %
Count
0
20
40
60
10
30
100
300
F-statistic (Log Scale)

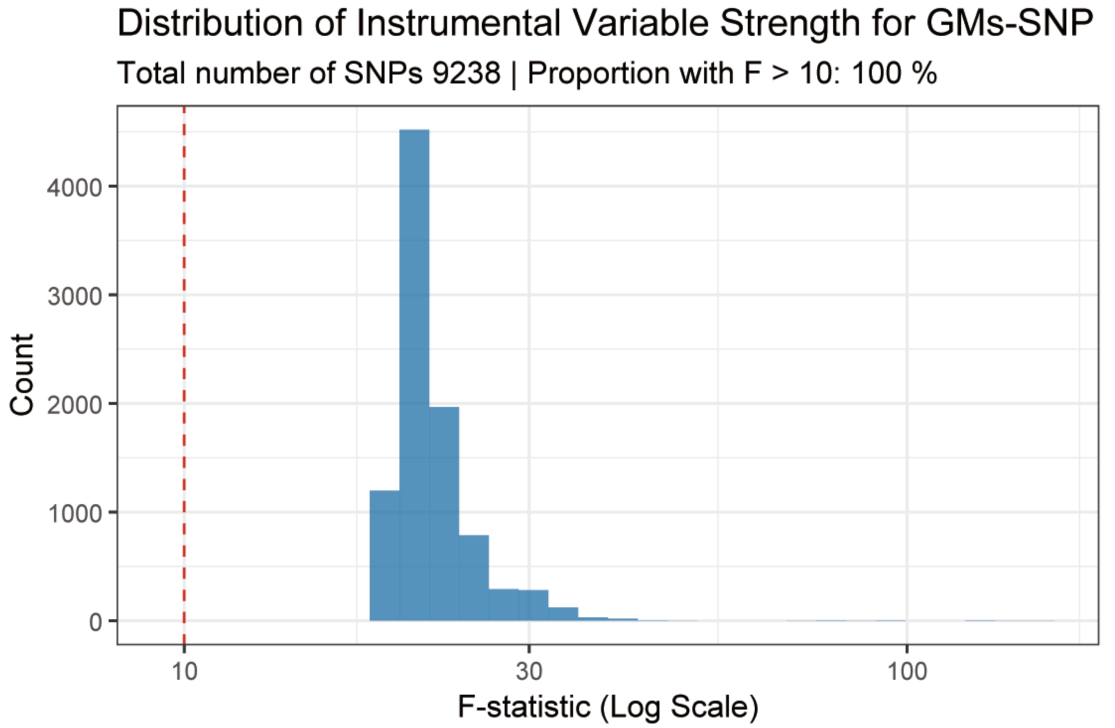
Distribution of Instrumental Variable Strength for GMs-SNP
Total number of SNPs 9238 | Proportion with F > 10: 100 %
Count
0
1000
2000
3000
4000
10
30
100
F-statistic (Log Scale)

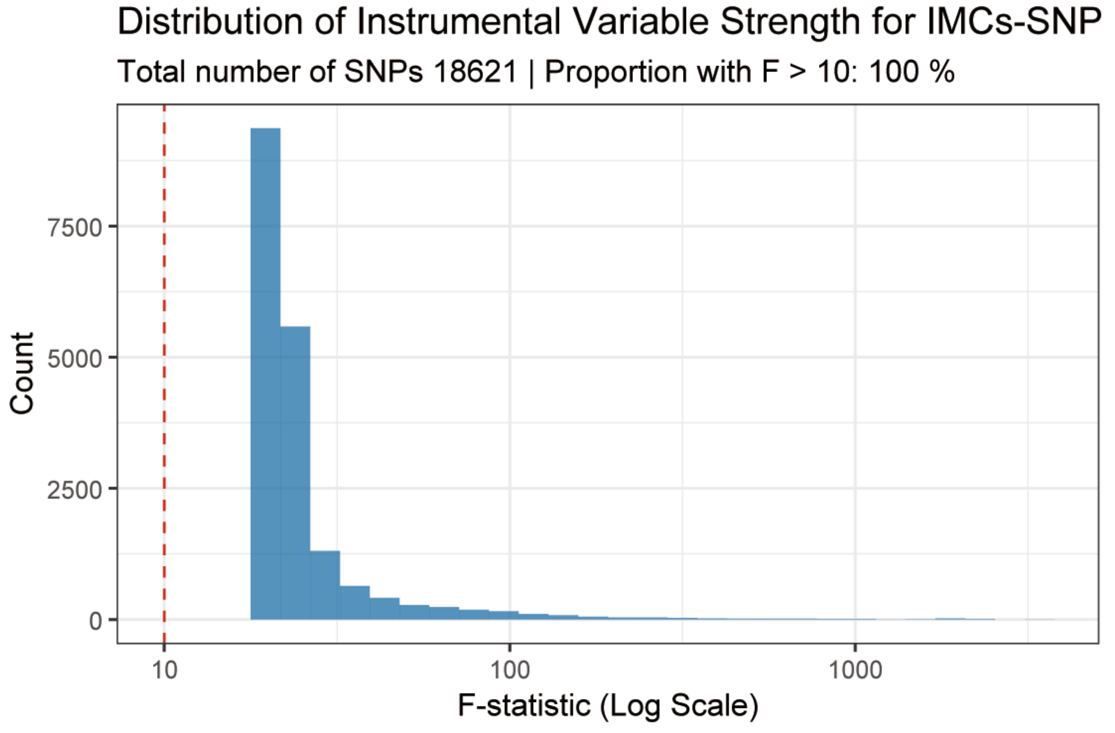
Distribution of Instrumental Variable Strength for IMCs-SNP
Total number of SNPs 18621 | Proportion with F > 10: 100 %
Count
0
2500
5000
7500
10
100
1000
F-statistic (Log Scale)
